# Calreticulin Release at an Early Stage of Death Modulates the Clearance by Macrophages of Apoptotic Cells

**DOI:** 10.3389/fimmu.2017.01034

**Published:** 2017-08-23

**Authors:** Rim Osman, Pascale Tacnet-Delorme, Jean-Philippe Kleman, Arnaud Millet, Philippe Frachet

**Affiliations:** ^1^University Grenoble Alpes, Institut de Biologie Structurale (IBS), CNRS, CEA, Immune Response to Pathogens and Altered Self (IRPAS) Group, Grenoble, France; ^2^ATIP/Avenir Team Mechanobiology, Immunity and Cancer INSERM U1205, Université Grenoble Alpes, Grenoble, France

**Keywords:** calreticulin, C1q, apoptotic cells, macrophage polarization, CD14, efferocytosis

## Abstract

Calreticulin (CRT) is a well-known “eat-me” signal harbored by dying cells participating in their recognition by phagocytes. CRT is also recognized to deeply impact the immune response to altered self-cells. In this study, we focus on the role of the newly exposed CRT following cell death induction. We show that if CRT increases at the outer face of the plasma membrane and is well recognized by C1q even when phosphatidylserine is not yet detected, CRT is also released in the surrounding milieu and is able to interact with phagocytes. We observed that exogenous CRT is endocytosed by THP1 macrophages through macropinocytosis and that internalization is associated with a particular phenotype characterized by an increase of cell spreading and migration, an upregulation of CD14, an increase of interleukin-8 release, and a decrease of early apoptotic cell uptake. Importantly, CRT-induced pro-inflammatory phenotype was confirmed on human monocytes-derived macrophages by the overexpression of CD40 and CD274, and we found that monocyte-derived macrophages exposed to CRT display a peculiar polarization notably associated with a downregulation of the histocompatibility complex of class II molecules hampering its description through the classical M1/M2 dichotomy. Altogether our results highlight the role of soluble CRT with strong possible consequences on the macrophage-mediated immune response to dying cell.

## Introduction

It is now unambiguously demonstrated that the multifunctional protein calreticulin (CRT) plays an important role in the phagocytic removal of apoptotic cells (1) when exposed at their surface and accordingly in the immune response driven by the phagocyte (2): first acting as an engulfment signal stimulating the recognition of dying cells by phagocytes (3) and second as an element of the antigen presentation machinery of macrophages and dendritic cells (DCs). It is now recognized, however, that CRT and more precisely cell surface-anchored CRT (that we will call ecto-CRT) interacts and collaborates with other molecules known to impact the process of elimination of cells dedicated to die. Nevertheless, CRT appears to play a role on both sides of the phagocytic synapse (also named efferocytic synapse) as it is also expressed at the surface of the phagocyte membrane. Some of CRT’s partners that modulate the whole efferocytosis process include other “eat-me” or “don’t eat-me” signals, bridging or/and signaling molecules and endocytic receptors. These molecules are C1q (bridging molecule), phosphatidylserine (PS; “eat-me” signal), CD47 (“don’t eat-me” signal), CD91/LRP (CRT receptor), and the scavenger receptor SRF-I (also called SREC-I or SCARF1) [reviewed in Ref. (4, 5)]. Interestingly some of these molecules have been shown to interact together, suggesting that the CRT-mediated immune response could be regulated by complex multimolecular interactions.

For example, we and others (6–8) have demonstrated that CRT interacts with the emblematic eat-me signal PS and also binds to the complement C1q protein, a bridging molecule that is also a PS-binding protein. CRT was early described as a C1q co-receptor with CD91 at the surface of macrophage even if the role of this interaction needs to be clarified (9, 10) and to bind the SRF-I endocytic receptor (11). Most strikingly, Ramirez-Ortiz et al. (12) have discovered that SRF-I on DCs, macrophages, and endothelial cells interacts with C1q-opsonized apoptotic cells and importantly that SRF-I-mediated uptake of apoptotic cells requires C1q and PS exposure on dying cells.

As it is noteworthy that ecto-CRT is capable of eliciting pro-inflammatory, immunogenic, signals in response to proapoptotic drugs used on cancer cells (13–16), the knowledge that several of its partners such as PS, C1q, and SRF-I have a pivotal role in the maintenance of immune tolerance and prevention of immune diseases (12, 17–19) added evidences that interplay of these molecules should be a key factor to balance the immune response to apoptotic cells.

Calreticulin amounts significantly increase at the surface of cells triggered to apoptosis, thus modulating the process of their elimination. Moreover, this protein is also found in the surrounding “efferocytic milieu” and at the surface of macrophages. CRT on macrophage has been described as a product of the macrophage (2, 20) but obviously could also result from soluble CRT adsorption (i.e., CRT released from dying cells). Thus far, however, little is known about the balance between endogenous and exogenous CRT anchored at the macrophage surface and their role(s) during efferocytosis.

We have previously analyzed the role of ecto-CRT on HeLa apoptotic cells uptake by THP1 macrophages (7, 21) and demonstrated that CRT and C1q interact at the cell surface, playing together to modulate not only the uptake process but also signaling events. We observed indeed that a deficiency in CRT induces contrasting effects on cytokine release by THP1 macrophages, by increasing interleukin (IL)-6 and monocyte chemotactic protein 1/CCL2 and decreasing IL-8. Remarkably, these effects were greatly reduced when apoptotic cells were opsonized by C1q, which counterbalanced the effect of CRT deficiency. It allowed to stress the role of the CRT–C1q interaction in this process and thereby to hypothesize that CRT effects are sharply delineated by its partnerships.

The present study focuses on the role of the newly exposed CRT following cell death induction using UVB irradiation. We developed a model of non-adherent cells that are harvested by simple centrifugation steps that facilitate the analysis of membrane-associated extracellular proteins. Here, we show that if ecto-CRT content increases before PS, the emblematic marker of apoptosis, can be detected, it is rapidly released in the medium. We investigated the functional consequences of this early release of CRT by apoptotic cells on macrophages. Notably, we observed that exogenous CRT is endocytosed by THP1 macrophages and that internalization, in turn, triggers a pro-inflammatory response. To better understand the activation state of macrophages resulting from the CRT exposition, we analyzed several surface markers associated with different polarization of human macrophages. The resulting phenotype of macrophages is associated with the secretion of the chemokine IL-8, an increase in the migration speed, and a decrease in the apoptotic cells phagocytosis ability, together with a decrease in the expression of the major histocompatibility complex of class II. These results highlight the profound consequences of the release of CRT by early dying cells, acting as a “chaperokine” (22), on the immune system.

## Materials and Methods

### Ethics Statement

Human blood samples from healthy donors (buffy coat) were obtained from Etablissement Français du Sang under the authorization number (CODECOH DC-2015-2502) with a signed consent.

### Cell Culture, Apoptosis Induction, and Macrophage Differentiation

JurkaT and THP1cells were cultured in RPMI-1640 medium (Invitrogen). J774 cells were grown in DMEM medium (Invitrogen). Media were supplemented with 10% heat-inactivated fetal calf serum (FCS) (v/v), penicillin (3 U/ml), and streptomycin (3 µg/ml). All cell lines were maintained at 37°C, under a 5% CO_2_ atmosphere. Apoptosis of JurkaT cells was induced in 12-well plates (2 × 10^6^ cells/ml; 1 ml/well) by UVB irradiation (1,000 mJ/cm^2^) at 312 nm in a fresh RPMI medium. Cells were then incubated for various times at 37°C. Apoptotic state of cells was assessed by flow cytometry using the Annexin V-FITC Kit (MACS MiltenyiBiotec) according to the manufacturer’s instructions. THP1 monocytes were differentiated into macrophage-like cells by treating with 10 nM PMA for 48 h (23, 24). Cell surface CRT was analyzed on THP1 macrophages incubated with soluble CRT or conditioned medium from JurkaT cells supernatants as follows: supernatants of JurkaT cells (2 × 10^6^ cells/ml) were collected by centrifugation at 320 *g*, 120 min after UVB irradiation. The conditioned medium of non-irradiated control cells was collected in parallel after 2 h. Wells of 12-well plate containing 0.5 × 10^6^ cells of THP1-derived macrophages were washed twice with RPMI medium, and JurkaT cells conditioned medium was added for 1 h at 37°C. For incubation with purified soluble CRT, 1 × 10^6^ THP1 cells differentiated with PMA for 48 h were incubated with CRT (1–6 µg/ml) in the presence of PMA for additional 24 h. Surface CRT was then analyzed by flow cytometry. For effect on CD14 expression, the dose of 6 µg/ml was determined experimentally (preliminary study). For control experiments, THP1 cells were cultured in the presence of polymyxin B (5 µg/ml) to avoid the effect of a potential LPS contamination. Peripheral blood mononuclear cells (PBMCs) were obtained from whole blood by density gradient centrifugation (Histopaque 1077 from Sigma-Aldrich). Monocytes were sorted with CD14 magnetic beads (Miltenyi Biotec) from PBMCs according to manufacturer instructions. Purity was assessed by flow cytometry for CD14^hi^CD45^hi^ cells and was >98%. Monocytes were cultured in RPMI-Glutamax supplemented with 10% heat-inactivated human serum AB (Sigma-Aldrich), and differentiation was obtained by M-CSF (Miltenyi Biotec) at 25 ng/ml or GM-CSF (Miltenyi Biotec) at 25 ng/ml during 5 days. Polarization was then obtained by changing the previous media with M-CSF adding CRT at 1, 3, or 6 µg/ml or vehicle (Tris 20 mM, pH 8, NaCl 150 mM, CaCl2 5 mM) or with GM-CSF during 60 h.

### Proteins

C1q and gC1q were purified from human serum and were prepared as previously described (21). Recombinant human CRT was expressed as *N*-ter HAT-tagged protein in Rosetta 2 cells (DE3) and purified by nickel-Sepharose chromatography as detailed in Ref. (25). CRT purified from human placenta (26) was provided by Gunnar Houen, Statens Serum Institut, Copenhagen, Denmark. To test endoxine contamination, CRT sample was digested by pronase (Sigma-Aldrich) 2 mg/ml in 50 mM Tris, 150 mM NaCl, 2 mM CaCl_2_, pH 7.4 for 24 h at 37°C, and then pronase was heat inactivated before incubation with cells. Purified CRT was labeled with Alexa fluor 488 (A488) by using Alexa Fluor Protein labeling Kit (Invitrogen) according to the kit’s instructions.

### Analysis of CRT Present in JurkaT Supernatants

Supernatants of 2 × 10^6^ JurkaT cells incubated in RPMI medium without serum were recovered at different times after irradiation (30 or 120 min). Cell supernatants were isolated by centrifugation at 320 *g* for 5 min for further analysis, and an equal volume of trichloroacetic acid (50% w/v in water) was added to each supernatant for one night at 4°C to precipitate contained proteins. Samples were then assessed for their CRT content by western blotting. Proteins were subjected to SDS-PAGE and transferred to nitrocellulose membrane that was then saturated with 5% milk in PBS-Tween solution. Reversible Ponceau red staining was routinely used as a loading control. Mouse anti-CRT monoclonal antibody SPA-601 (1 µg/ml) was added as a primary antibody. For signal detection, horseradish peroxidase-conjugated secondary antibody was used, and the chemiluminescence of bands was revealed using the Amersham ECL Selected western blotting detection reagent (GE Healthcare) on ChemiDoc MP Bio-RAD system.

### Endocytosis Analysis

To measure exogenous CRT ingestion, THP1 macrophages were incubated with 10 µg/ml of CRT conjugated with A488 for 1 h at 37°C. For inhibitions, cells were preincubated before fluorescent CRT addition for 30 min at 37°C with the following inhibitors: 20 µM cytochalasin D (Sigma-Aldrich), 100 µM 5-(*N*-Ethyl-*N*-isopropyl)amiloride (EIPA) (Sigma-Aldrich), or 150 mM sodium azide NaN3 (Sigma-Aldrich). Cells were then harvested with 0.25% trypsin–EDTA, resuspended in PBS, and directly analyzed by flow cytometry.

### Wound Healing Scratch Assay

J774 were cultured in 12-well dishes at 10^6^ in 1 ml of DMEM with FCS. After 24 h, cells were washed with medium, and wounds were drawn in each well by mechanical scarping using a 200-µl pipette tip. Displaced cells were eliminated by washing, and 1 µg/ml of CRT was added. Transmitted light micrographs were immediately taken (T0) and 40 h after CRT addition (T40) with an inverted light microscope. Areas of the scratch at these two time points were measured using Image J software, and the percent of wound closure was calculated according to the formula: normalized area of scratch at T0 divided by the normalized area of the scratch at T40.

### Cell Spreading Analysis

THP1 were differentiated into macrophages in six-well culture plates at 10^6^/well; 3 ml of RPMI with FCS. After 48 h, cells were washed in PBS, and 3 ml of fresh RPMI medium containing 1 µg/ml of CRT was added for 24 h. Following washing, wells were viewed with an inverted light microscope, and three images were taken in three separate zones. The percentage of spread cells was calculated by counting the number of cells with spread shape in a total of 300 cells (100 per zone) for each condition.

### Flow Cytometry Analysis

Double-staining PS/ecto-CRT of JurkaT cells was performed by using Annexin V-FITC Kit (MACS Miltenyi Biotec) and chicken anti-CRT antibody PA1-902A (Thermo Scientific). For this, cells collected 30 or 120 min after UVB irradiation were first stained for PS by Annexin V-FITC. Cells were then washed in PBS, fixed for 7 min with 4% paraformaldehyde (PFA) at room temperature, and saturated with PBS-1% BSA for 15 min. Both anti-CRT antibody or chicken isotype IgY control (Jackson ImmunoResearch) at 5 µg/ml in the saturation solution were added for 45 min at RT. Cells were then washed twice in PBS before incubation in the dark for 30 min with cyanine-3-conjugated donkey anti-chicken Ig secondary antibody (Jackson ImmunoResearch, 1/200). Cells were finally washed and suspended in PBS before flow cytometry analysis. To control the percentage of necrotic permeabilized cells in the whole population, staining with propidium iodide (1 µg/ml) was established for each condition. For THP1 labeling, cells were washed twice with PBS and harvested with 0.25% trypsin/EDTA solution. Cells were fixed in PFA and stained for ecto-CRT as described above. CD14 labeling of THP1 (anti CD14-PE, clone RMO52, Beckman Coulter) was performed without fixation. For labeling of macrophages obtained from monocytes, antibodies used were CD40 FITC (clone HB14), CD14 FITC (clone TÜK4), CD206 PE (clone DCN228), CD74 APC (clone 5-239), CD274 APC (clone MIH18), HLA-DR APC (clone AC122), and the corresponding isotypes (Miltenyi Biotec). Cells doublets were gated out by comparison of forward scatter signal height and forward scatter signal area. Dead cells were eliminated with 7AAD or FSC/SSC profile. For all experiments, FcR blockade was performed with the FcR-blocking solution (Miltenyi Biotec). Flow cytometry analyses were performed with MACSQuant VYB cytometer (Miltenyi Biotech, M4D cell imaging platform, IBS), and collected data were treated with MACSQuantify software or with an Accuri C6 (Becton Dickinson) flow cytometer. A least 10,000 events in the analysis gate were collected.

### Fluorescence Microscopy and Fluorescence Resonance Energy Transfer (FRET) Quantification

Fluorescence resonance energy transfer experiment was adapted from our previous published protocol (21). JurkaT cells were directly seeded in eight-well glass slide Millicell EZ (Merck Millipore) at a concentration of 0.3 × 10^6^ in 300 µl of RPMI without FCS, irradiated as described above, and incubated for 30 min at 37°C. During our work with JurkaT cells, we noted that the incubation of these cells in medium without FCS induces their adherence to the culture plate. This may be due to the absence of factors probably contained in FCS. Cells were washed with PBS, fixed for 7 min with 4% PFA, saturated in PBS-1% BSA for 15 min at RT, and incubated for 30 min at RT with gC1q or C1q at 80 µg/ml in the saturation solution. Cell surface CRT and bound gC1q were indirectly immunodetected by using primary antibodies (PA1-902A chicken polyclonal anti-CRT antibody (5 µg/ml) and a rabbit polyclonal anti-gC1q diluted 1/200) and secondary antibodies (Alexa 488-conjugated donkey anti-chicken IgY and cyanine 3-conjugated goat anti-rabbit IgG, respectively). Cells slides were mounted glass slides using Vectashield solution with 4’,6’-diamidino-2-phenylindole (Vector Laboratories) and were visualized under a laser spinning-disk confocal microscope (Olympus & Andor, M4D cell imaging platform, IBS). FRET signal was measured by performing acceptor fluorescence photobleaching (cyanine-3) on zones where CRT and gC1q co-localize with a measured Pearson coefficient located between 0.7 and 1. The FRET efficiency is calculated as previously detailed (21). Data were evaluated with Volocity software. Control experiments were performed using primary and secondary antibodies in the absence of gC1q and secondary antibodies alone in the presence of gC1q. For actin visualization, THP1 macrophages incubated for 24 h at 37°C in the presence or absence of 1 µg/ml CRT were fixed with 4% PFA, permeabilized in PBS–0.1% Triton for 10 min at RT and stained with fluorescent phalloidin-TRITC (Sigma). Cells were observed with epifluorescence microscope (Olympus & Perkin-Elmer, M4D cell imaging platform, IBS).

### Quantification of Cytokine Release

Supernatants of THP1 macrophages treated 24 h with CRT as mentioned above were collected and then analyzed for IL-6 and IL-8 contents by ELISA according to manufacturer instructions (Covalab).

### Phagocytosis Assay

JurkaT cells were labeled with CFSE (CellTrace™ CFSE Cell Proliferation Kit, Life Technologies) as follows: cells were washed twice and then resuspended at 1 × 10^6^ cells/ml in PBS and incubated with 1 µM CFSE at 37°C for 20 min. The remaining CFSE was quenched with addition of RPMI-10% FCS for at least 10 min. Cells were then pelleted by centrifugation and resuspended in RPMI-10% FCS before the induction of apoptosis. Apoptotic JurkaT cells were then added to THP1-derived macrophages that had been preincubated or not with CRT, at a ratio of 10:1 (JurkaT:THP1), for 1 h at 37°C, 5% CO2. After incubation, cells were washed and harvested with 0.25% trypsin/EDTA and then labeled for CD14 as described above for 15 min at room temperature and immediately analyzed by flow cytometry. Phagocytosis was calculated as the percentage of the double CFSE and CD14-PE-labeled cells in the THP1 macrophage population (CD14-PE-positive cells). Significance was tested using non-parametric Wilcoxon signed-rank test for paired samples.

## Results

### Ecto-CRT Increases Rapidly after UVB Irradiation and before PS Exposure and Then Is Released in the Surrounding Milieu

**Figure 1:**
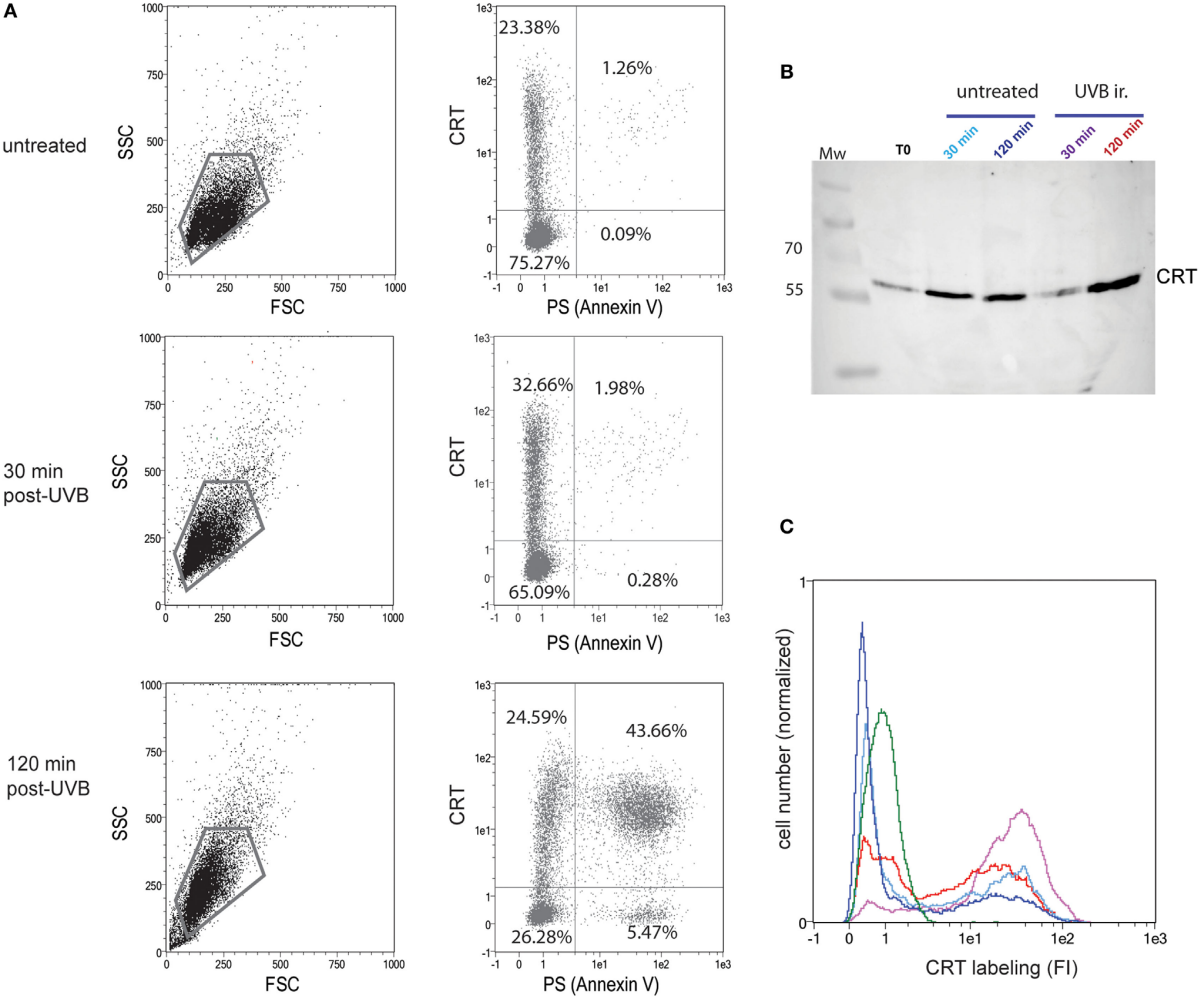
Calreticulin (CRT) is exposed rapidly after UVB irradiation at the cell surface and released in the medium. **(A)** Uuntreated or UVB-irradiated JurkaT cells were analyzed by flow cytometry for their phosphatidylserine (PS) (Annexin V-FITC) and CRT (anti-CRT antibody/secondary antibody-Cy3) surface exposure. The region selected for double PS/CRT analysis is shown on SSC/FSC dot plots. Double PS/CRT labeling dot plots: % of cells in quadrants is indicated. **(B)** CRT detection by western blotting on the medium conditioned by JurkaT cells and collected at different times as mentioned. Each line corresponds to 1 ml of medium conditioned by 2 × 10^6^ cells. T0 point corresponds to medium added to untreated cells and immediately recovered. Mw, molecular weight ladders (kilodaltons). **(C)** In parallel to supernatants recovering, cells used for the analysis shown in B were collected, immediately fixed, and labeled for surface CRT detection by flow cytometry. Ungated populations were analyzed. B and C, The same color code is used for conditioned medium or the corresponding cells: untreated control condition after 30 min of incubation (light blue) or 120 min (dark blue), UVB-irradiated condition after 30 min of incubation (purple) or 120 min (red). An isotype control is shown in green. **(A–C)** Representative experiments are shown.

One of our primary interests was to follow the dynamic CRT exposure following the death induction. For this, we settled a dying cell model with the non-adherent JurkaT cell line that allows us to efficiently access cell surface, i.e., without scraping the cells that could alter the cell membrane. We thus verified that whether under UVB radiation, a known inducer of apoptosis, CRT expression at the outer leaflet of the plasma membrane is increased (Figure 1). We confirmed, by double Annexin V/anti-CRT cell surface labeling, that CRT increased significantly 30 min after UVB treatment at the cell surface even though PS translocation was still not detected (Figure 1A; untreated and 30 min post-UVB conditions), in agreement with previously published data (27). After 120 min post-UVB treatment, when apoptosis is characterized by a huge increase of PS-positive cells, more than 90% of the PS-positive cells also expose ecto-CRT at their surface. Interestingly the observation that the portion of ecto-CRT-positive cells measured at 30 min becomes PS positive at 120 min strongly suggests that CRT translocation to the membrane could be an initial and potentially a required step, for apoptosis development under the UV stimulus.

Because we hypothesized that ecto-CRT should pull away from the membrane in the surrounding medium, we next analyzed the CRT contents of the cell medium, in parallel to CRT membrane-exposure after UVB irradiation. In this experiment, JurkaT cells were UVB irradiated in a serum-free medium, and extracellular CRT was sensed from the cell supernatant by western blotting (Figure 1B) while the corresponding pellet of cells were surface labeled with an anti-CRT antibody (Figure 1C). As observed by immunoblotting, CRT was detected in the cell medium even for untreated population and also recovered by a simple step of washing (T0), thus suggesting that ecto-CRT is not strongly anchored to the membrane. After UVB irradiation, extracellular CRT was at first significantly reduced (30 min postirradiation) when compared to the control condition (untreated 30 min) and then notably released (at 120 min). Of interest, when the corresponding cells were analyzed by flow cytometry, we observed that cell population analyzed 30 min after UVB irradiation presented a more marked CRT positive part (purple curve) than irradiated cells recovered at 120 min (red curve) or than untreated cells. Altogether these observations mainly showed that the membrane of early/preapoptotic cells are firstly enriched for ecto-CRT and then that this CRT is released in the medium.

### Early Exposed–CRT Co-Localizes and Interacts with C1q

**Figure 2:**
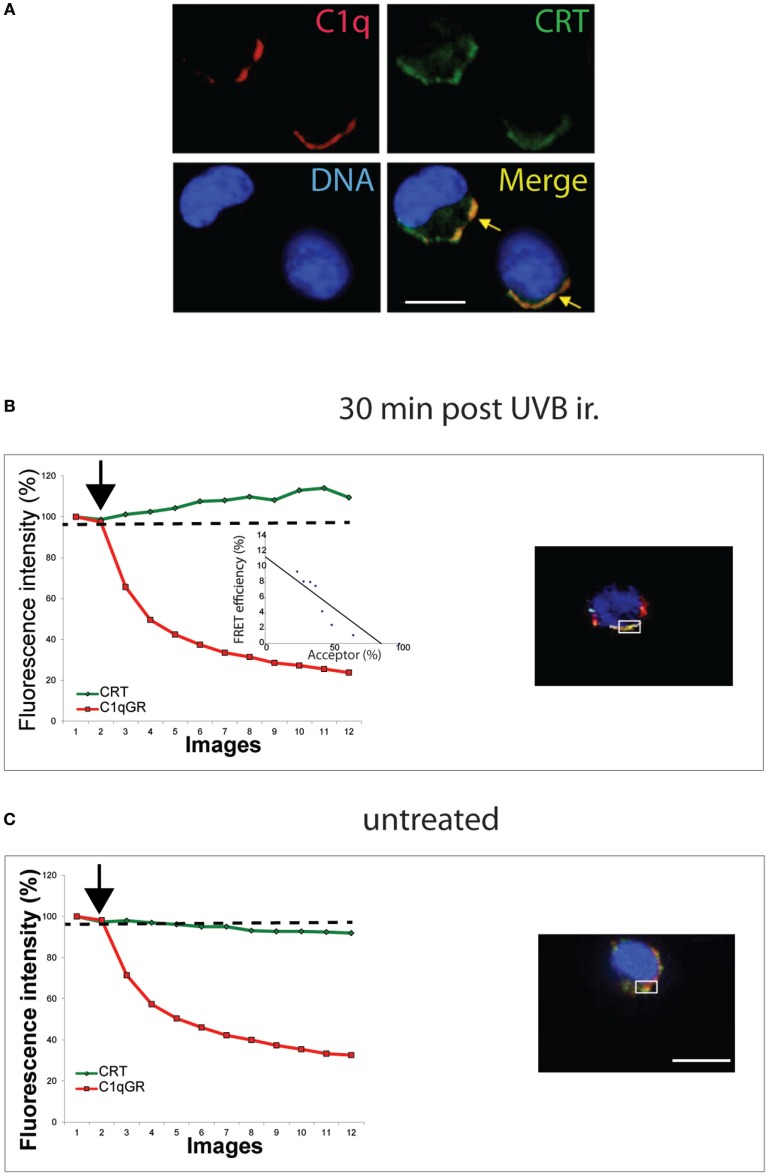
C1q interacts with ecto-calreticulin very rapidly after UVB irradiation. JurkaT cells incubated with C1q or its globular region were immunolabeled for Calreticulin (CRT) (A488) and C1q or C1qGR (Cy3) as described in Section “Material and Methods.” **(A)** Labeling on cells fixed 30 min after UVB irradiation. **(B,C)** Fluorescence resonance energy transfer (FRET) efficiency was estimated for UVB-irradiated cell and untreated cell by photobleaching of the acceptor dye (Cy3) on non-permeabilized cells. Regions used for the acceptor photobleaching and FRET analysis are shown. Curves correspond to the normalized fluorescence intensities of both dyes (Cy3 in red and A488 in green) expressed as a percent of the signal measured before the gradual photobleaching started (black arrow). **(B)** FRET efficiency (percent of acceptor fluorescence intensity increase) is expressed as a function of the percent of the normalized acceptor fluorescence intensity. Bars: 11 µm.

To determine if this CRT could impact the recognition of this early/preapoptotic UVB-treated cells, we next performed co localization imaging with the complement protein C1q, characterized previously to cooperate with CRT in the uptake of apoptotic HeLa cells (21). As shown in Figure 2A, C1q and CRT co-localize at the surface of JurkaT cells, fixed, and labeled 30 min after the UVB irradiation. Furthermore, we observed by FRET measurement that the globular head of C1q (gC1q) interaction with CRT occurs on this UVB-treated JurkaT cells (Figure 2B), whereas we are unable to measure any specific FRET signal on untreated cells even when apparent co localization was observed by confocal microscopy (Figure 2C). These data are in good agreement with our previous observation that C1q and CRT interaction occurred on PS-positive HeLa cells at early apoptotic stages, but not anymore after membrane blebs can be detected, despite the continuous co localization of CRT and C1q (21). Thus, the early exposure of ecto-CRT at the surface of JurkaT cells undergoing apoptosis appears readily accessible to C1q and probably to other physiological ligands, potentially modulating the immune response to apoptotic cells *in vivo*.

### CRT Released in the Culture Medium Binds to Macrophages and Is Endocytosed by a Macropinocytosis-Like Process

**Figure 3:**
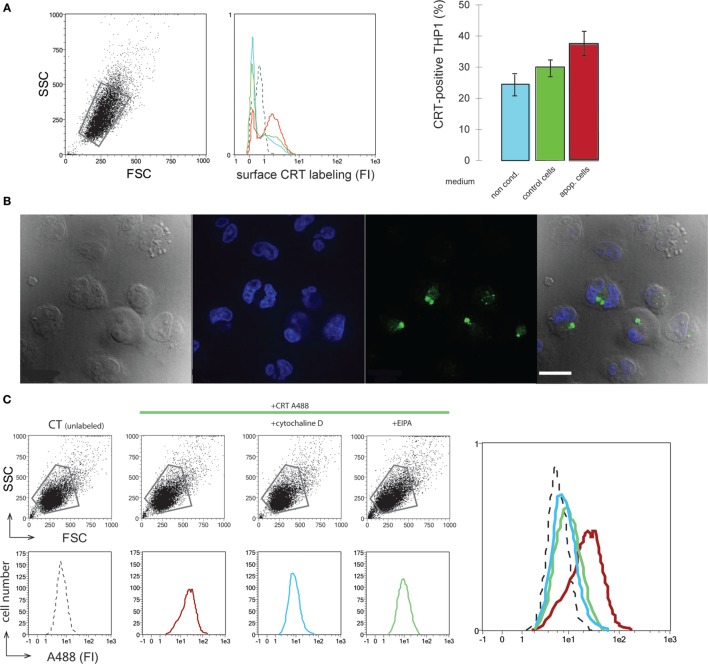
Soluble calreticulin (CRT) binds to phagocyte and is endocytosed. **(A)** THP1 macrophages were incubated with normal medium (blue), medium conditioned by control (green), or apoptotic (red) JurkaT cells. Surface CRT was detected by immunobaleling and flow cytometry analysis. FSC/SSC dot plot, fluorescence histograms, and the quantification of the CRT-positive cells in the corresponding gated population were shown. Isotype control is shown in dotted line (middle panel). **(B)** THP1 macrophages incubated with CRT-A488 were visualized by microscopy under differential interference contrast, DAPI, and A488 filters, merge is shown (left to right panels). Bar: 22 µm. **(C)** FSC/SSC dot plots and fluorescence histograms of the corresponding gated populations of THP1 macrophages incubated with CRT-A488 alone or in the presence of endocytic inhibitors as mentioned. An overlay of the data is shown. Unlabeled control cells (dotted line), CRT-A488 alone (red), CRT-A488 plus Cytochalasin D (light blue), and CRT-A488 plus 5-(*N*-Ethyl-*N*-isopropyl)amiloride (green).

The efficient release of CRT by dying cells leads us to examine the potential effects of extracellular CRT on the phagocyte. An initial approach was to analyze the effect of the medium conditioned by UVB-irradiated JurkaT cells, which contains the released CRT (Figure 1), on the macrophage. As shown in Figure 3A, THP1 macrophages when incubated with the UVB-irradiated JurkaT cells conditioned medium collected 120 min posttreatment (IR120 as referred on Figure 1, which contains the greater amount of CRT) are more labeled for CRT on their surface in contrast to those incubated with normal medium or medium conditioned by viable cells. This increase was measured for short incubation time (1 h), which indicates most likely that the extracellular CRT from the medium binds to the macrophage cell surface rather than it was due to newly expressed endogenous CRT. Further experiments were then conducted using recombinant CRT (rCRT) (7) to analyze its role(s) on the macrophage biology. Interestingly rCRT conjugated with Alexa 488 was shown to be efficiently ingested in large vesicles by the THP1 macrophages (Figure 3B). To characterize the endocytic route, we tested the effect of several endocytosis inhibitors. As expected, general inhibitors such as sodium azide (NaN3) (data not shown) or Cytochalasin D (Figure 3C) blocked the uptake of rCRT. Finally, EIPA or amiloride, a specific inhibitor of macropinocytosis (28), was shown to markedly inhibit the ingestion of the exogenous rCRT-A488 by the THP1 macrophages.

### Exogenous CRT Modulates Macrophage Spreading and Migration

**Figure 4:**
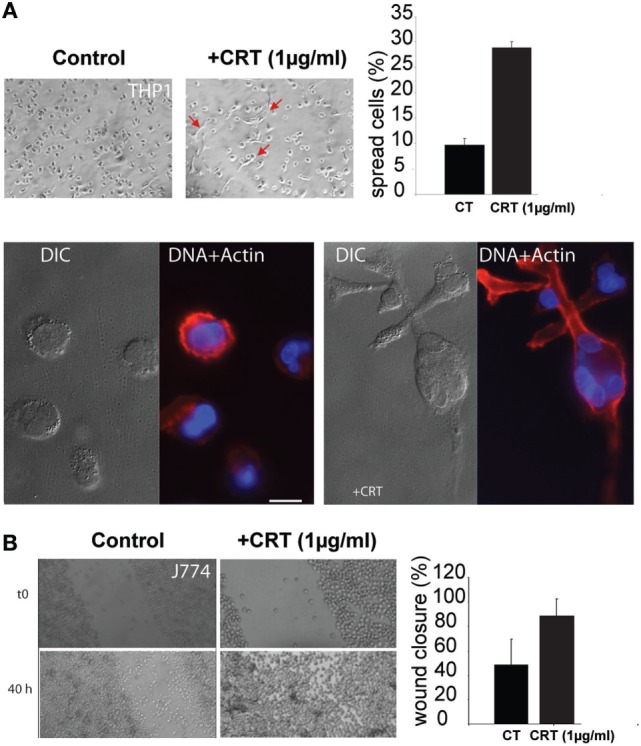
Soluble calreticulin (CRT) induces macrophages spreading and migration. **(A)** THP1 macrophages were treated with 1 µg/ml of recombinant CRT, or not, for 24 h, and cell spread was evaluated. The spreading phenotype was confirmed by actin fibers labeling with phalloidin-TRITC. Characteristic differential interference contrast (DIC) and fluorescent images (DAPI/TRITC) are shown. Bar: 10 µm. **(B)** Wound healing scratch assays were realized using the J774 mouse macrophage cell line, in the presence or absence of CRT. The closure efficiency was evaluated as described in Section “Material and Methods.”

To study the possible influence of the exogenous CRT (e.g., released by apoptotic cells) on the macrophage response, we incubated THP1 macrophages with rCRT. THP1 monocytes treated with PMA (to induce their differentiation into macrophages) appeared substantially more spread in the presence of 1 µg/ml of rCRT, and this was confirmed by actin-stained stress fibers showing characteristic phenotype (Figure 4A). As it was previously reported that CRT could impact migration of various cell types (29), we performed wound healing assays to analyze the rCRT effect on macrophages migration. Due to the inability of human THP1 macrophages differentiated with PMA to form a homogenous confluent layer, this assay was obtained using the mouse J774 adherent macrophage cell line. As illustrated on Figure 4B, 1 µg/ml CRT enhanced significantly the wound closure. This effect is independent on cell proliferation as we controlled that rCRT did not impact J774 proliferation (data not shown).

### Exogenous CRT Acts on Macrophages Phenotype

**Figure 5:**
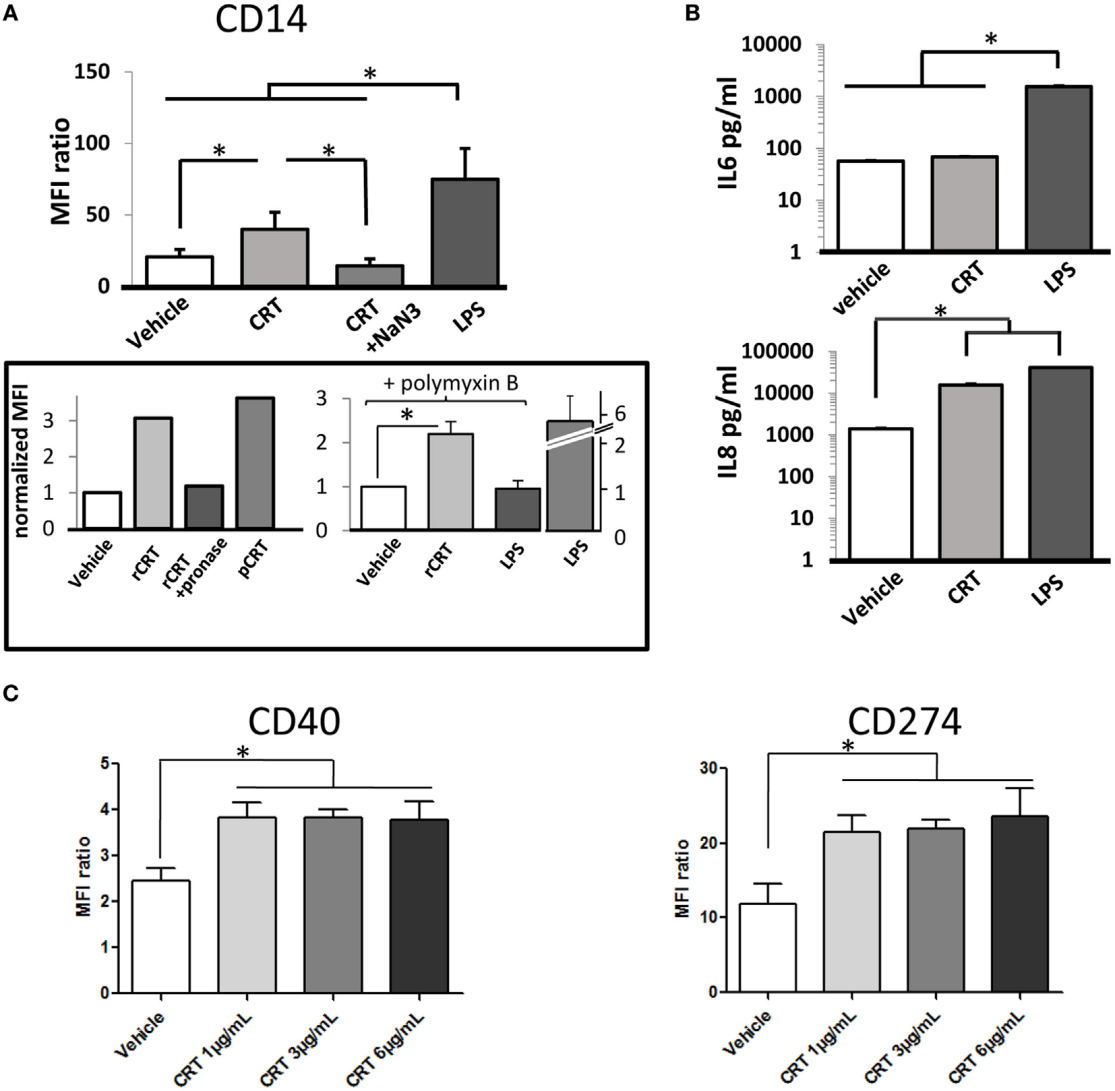
Activation of calreticulin (CRT)-exposed macrophages. **(A)** CRT increases the CD14 expression of the THP1 cells. MFI for CD14 labeling is shown for control PMA-treated THP1 (vehicle), incubated with CRT (6 µg/ml), CRT and sodium azide, or LPS (1 µg/ml) as a positive control (*p < 0.05, *n* = 6 independent experiments, Tukey’s multiple comparison test). Two independent controls are shown (box): comparison between recombinant CRT (rCRT) (6 µg/ml), pronase-treated rCRT, and placenta-isolated CRT (6 µg/ml); experiment in the presence of polymyxin B (5 µg/ml) to inhibit effect of potential LPS contamination on CD14 expression. The CD14 expression induced by LPS in the absence of polymyxin is shown (LPS alone); this effect is abolished by 5 µg/ml of polymyxin (LPS in the presence of polymyxin). CRT (6 µg/ml) and LPS (10 ng/ml) (*n* = 3, *p < 0.05) and MFI were normalized to the condition without recombinant CRT. **(B)** Interleukin (IL)-6 and IL-8 release by THP1 in the presence of CRT measured by ELISA (*p < 0.05, *n* = 3 independent experiments, Tukey’s multiple comparison test). **(C)** Expression of membrane proteins CD40 (*tnfrsf5*) and CD274 (*pdl1*) for human macrophages exposed to CRT at 1, 3, and 6 µg/ml compared to vehicle (*p < 0.05, Tukey’s multiple comparison test, *n* = 3 independent human donors). **(A,C)** Quantification were obtained by flow cytometry.

We next analyzed the effects of recombinant CRT on cytokines released by THP1 macrophages and on expression of macrophage differentiation markers. CD11b and CD11c expression (αM integrin and αX integrin subunits, respectively) are upregulated in response to PMA stimulation without any detectable effect of rCRT (data not shown). In contrast, adding rCRT enhanced CD14 expression in the THP1 population (Figure 5A). This increase of surface CD14 was almost half of what is obtained with LPS and was very efficiently inhibited by blocking endocytosis with sodium azide. As controls, pretreatment of recombinant CRT with pronase to degrade CRT protein abolished this effect; and in addition, a similar increase in CD14 expression was obtained using CRT purified from human placenta (Figure 5A). This ensures that our observation was not significantly affected by LPS endotoxin contamination. Accordingly, we reproduced CRT effect on CD14 expression in the presence of 5 µg/ml of polymyxin B (Figure 5A). We observed moreover that rCRT induces an increase of the secretion of IL-8 classically considered as a pro-inflammatory chemokine, whereas secretion of IL-6 is not significantly modified (Figure 5B). This pro-inflammatory phenotype is confirmed by the induction of the expression of CD40 (tumor necrosis factor receptor superfamily member 5) and CD274 (programmed cell death 1 ligand 1/PD-L1) on human monocyte-derived macrophages exposed to rCRT (Figure 5C). These surface markers are associated with a classical activation of macrophages, usually obtained by stimulation with IFN-γ and/or LPS (30, 31).

### CRT Triggers a Non-Conventional Macrophage Polarization and Impairs the Expression of MHC-II Molecules

**Figure 6:**
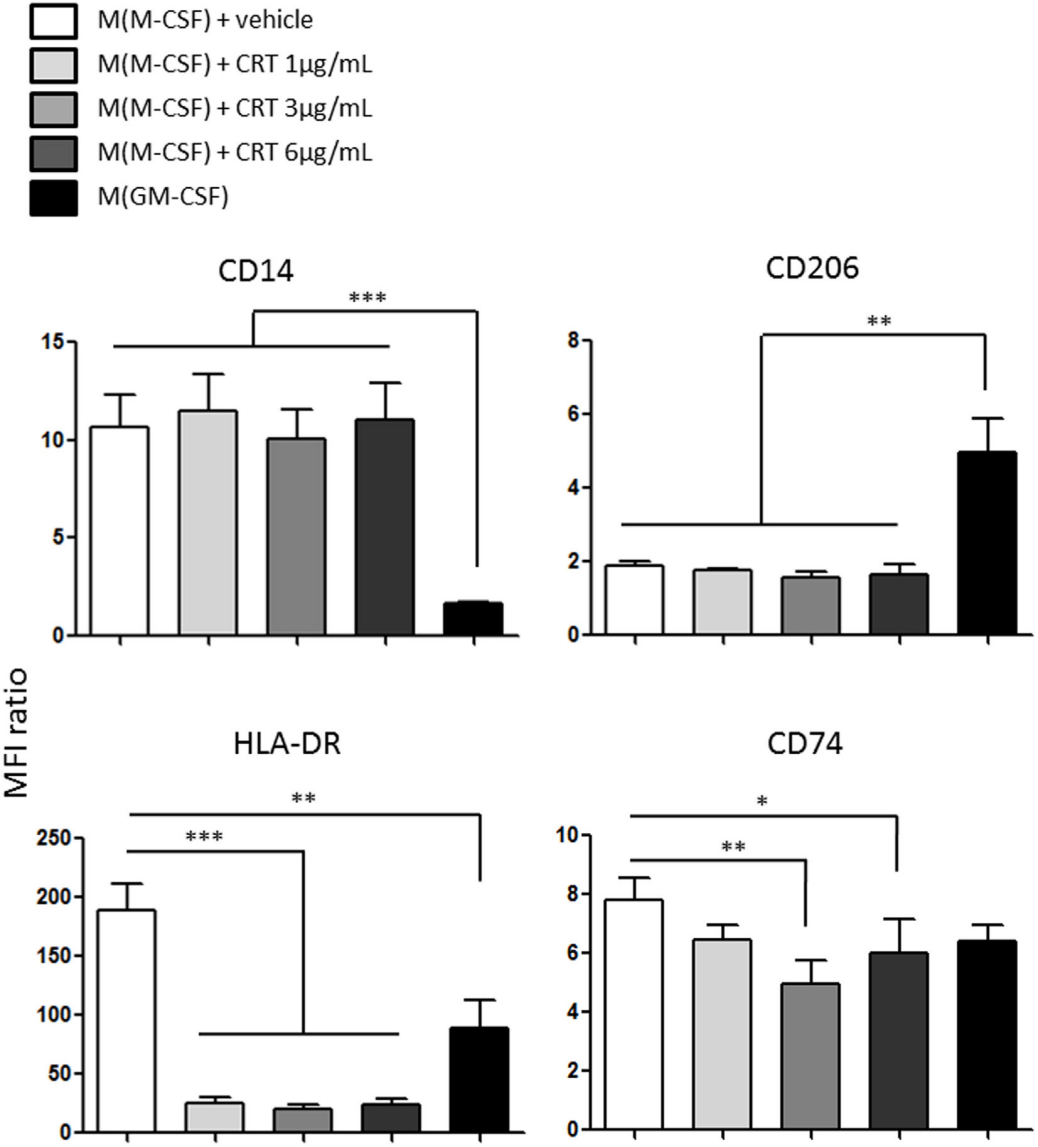
Polarization profile of human monocytes-derived macrophages exposed to calreticulin (CRT). Expression of membrane proteins obtained by flow cytometry for CD14, CD206 (*mrc1*), HLA-DR, CD74 (*dhlag*), and for human macrophages (differentiated with M-CSF) exposed to CRT at 1, 3, and 6 µg/ml or vehicle compared to macrophages differentiated with GM-CSF (*p < 0.05, **p < 0.01, ***p < 0.001, Tukey’s multiple comparison test, *n* = 3 independent human donors).

Human monocytes CD14hi-derived macrophages were polarized using M-CSF or GM-CSF. Their expression of surface markers like CD14, CD206 (Mannose receptor), and MHC-II molecules like HLA-DR and CD74 (HLA class II histocompatibility antigen gamma chain) was obtained by flow cytometry. We determined the expression of M-CSF differentiated macrophages (during 5 days) exposed to vehicle or increasing concentration of CRT (1, 3, and 6 µg/ml) during 60 h for these markers (Figure 6). If CRT does not modify the expression of CD14 and CD206, which is strongly differentially expressed between M(M-CSF) and M(GM-CSF) (Figure 6 upper panel), it completely downregulated the expression of HLA-DR and to a lesser extent the expression of CD74 (Figure 6, lower panel). This result is not usually found in classically activated macrophages M(LPS + IFN-γ), which express a stronger level of HLA-DR than M(M-CSF), which illustrates the peculiarity of the activation state induced by CRT on human macrophages and shows that M(ecto-CRT) macrophages do not fit with the traditional M1/M2 dichotomy.

### CRT Affects Engulfment of Apoptotic Cells

**Figure 7:**
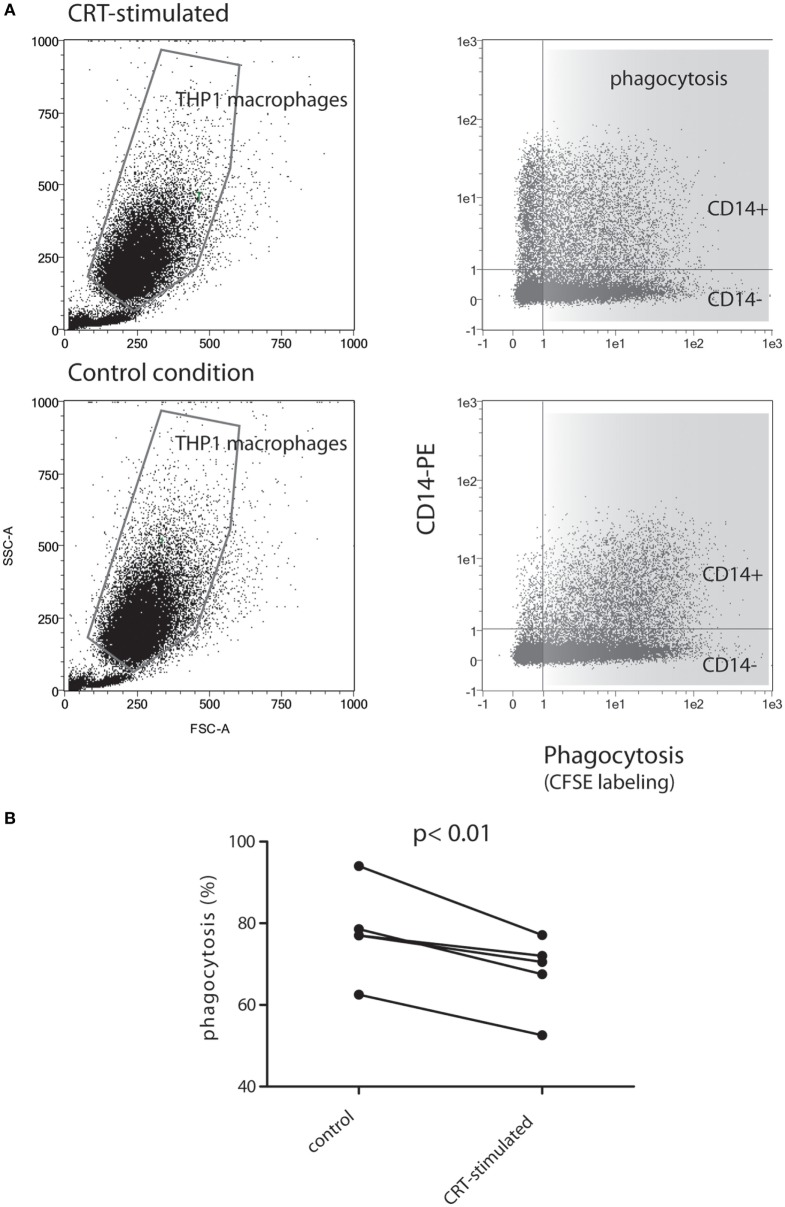
THP1 macrophages differentiated in presence of soluble calreticulin (CRT) decrease phagocytosis of early apoptotic cells. **(A)** CFSE-labeled JurkaT cells were UVB irradiated, incubated 2 h at 37°C, and then mixed with THP1 macrophages for phagocytosis assay as described in Section “Material and Methods.” Representative data are shown for THP1 differentiated in the absence or presence of CRT. Immediately after phagocytosis, cells were labeled with anti-CD14-PE and analyzed by flow cytometry. The gated macrophage population was analyzed for its double PE/CSFE labeling (right panels). **(B)** Quantification of phagocytosis from five independent experiments is shown. The result highlights a decrease of double-labeled macrophages (i.e., decrease of phagocytic events) for CD14^+^ THP1 macrophages treated with CRT compared to controls.

Assuming that polarization of macrophages could impact the phagocytosis process, we next tested the abilities of soluble rCRT to impact the capacity of THP1 macrophages to engulf early apoptotic cells. Early apoptotic JurkaT cells collected 2 h after UVB irradiation were subjected to THP1 macrophages pretreated or not by soluble recombinant CRT for 24 h. As shown in Figure 7, the CRT-treated population of macrophages (upper panels), which is enriched for CD14-expressing cells, decreases its efficiency to ingest apoptotic cells. Indeed, when exposed to CRT, 66 ± 9% (% of double labeled THP1 cells ± SD) of the CD14^+^ THP1 macrophages population engulfed early apoptotic JurkaT cells, instead of 78 ± 11% for the control, which correspond to a significant phagocytic efficiency decrease of 13.2 ± 3.5% (mean of five independent experiments ± SD, *p* < 0.01).

## Discussion

In this study, we demonstrated that cell surface CRT increases in the cell population rapidly after UVB irradiation and before the expression of PS at the outer face of the plasma membrane. As we have previously shown, CRT is a common PS and C1q partner and could modulate C1q binding to apoptotic cell surface through a possible interference with the PS–C1q interaction (7). We have tested the C1q interaction with surface-exposed CRT at the early stages of apoptosis, when PS is not yet detected. Herein, we were able to show, by FRET measurements, that CRT and C1q bind together. This argues in the favor of a physiological function of CRT-dependent C1q opsonization at the very beginning of the programmed cell death process, even though its real effect, either on apoptotic cell uptake or on phagocyte signaling events, remains to be studied in detail. We then observed that ecto-CRT, which appears to initially increase at the cell surface, is secondarily released in the surrounding milieu and could bind to the surface of neighboring macrophages. To understand the potential effect of soluble CRT on macrophages, we showed that on THP1 macrophages, the recombinant CRT is efficiently endocytosed by a macropinocytosis-like process. This internalization was associated with (i) an increase secretion of a pro-inflammatory chemokine (IL-8), (ii) an increase in cell spreading and cell migration as it has been already reported (29), and (iii) an upregulation of the expression of CD14 molecules. Importantly, the CRT-induced pro-inflammatory phenotype was confirmed on human monocytes-derived macrophages by the overexpression of CD40 and CD274. If our results point toward a pro-inflammatory activation of macrophages, CRT-induced polarization displays a peculiar expression of surface markers with strong possible consequences on the immune system. We found that the expression of HLA-DR, which is deeply involved in the presentation of antigens, and to a lesser extent CD74, also a MHC-II molecule that helps guiding the CD74–MHC-II complex move on to the endolysosomal pathway, are downregulated by the exposition to CRT. This result suggests that macrophage exposed to CRT could have an antigen presentation mechanism by the MHC-II pathway impaired leading to a decreased ability to recruit and activate helper CD4^+^ T cells. Because CRT enhances the stabilities of components of the MHC class I assembly pathway (30), it is tempting to suggest that CRT could favor the use of a cross presentation as an antigen presentation mechanism for macrophages.

Altogether our results suggest that CRT mainly intracellular on viable cells, rapidly exposed on apoptotic cell surface and efficiently released is a significant “inducing signal” for macrophages, able to modulate macrophage polarization. In support to this proposition, Bajor and collaborators (22) have previously demonstrated that CRT induced the maturation of human DCs and increases the production of pro-inflammatory cytokines. Of note, this DC maturation was found particularly clear when the conditioned medium obtained by monocytes exposed to CRT was used. This conditioned medium was able to induce a strong maturation of DCs associated with CD83, CD86, and HLA-DR overexpression and secretion of pro-inflammatory factors like IL-6, IL-8, and TNF-α. The hypothesis proposed for this effect is the activation through TLRs of the NF-kB pathway induced by released factors present in the conditioned medium (22). It has been reported that murine CRT is unable *in vitro* and *in vivo* to induce the maturation of DCs contrary to what is found with human cells (32). The mechanism by which CRT could directly activate human macrophages is not known and will need further investigations.

Our finding that soluble CRT can be internalized through a macropinocytosis-like process, together with the observed effect of CRT on HLA-DR expression on monocyte-derived macrophages add clues for a potential role of CRT in the regulation of antigen presentation. Given that phagocytosis is tightly dependent on macrophages polarization and because CD14 expression ties to macrophage differentiation, we tested the CRT-treated THP1 macrophages for their ability to engulf apoptotic cells. Interestingly, we noted a significant CD14-dependent decrease of the phagocytosis events when CRT-treated and CRT-untreated THP1 macrophages were compared. This should also account for a more efficient polarization into M1-like type macrophages, less able to eat altered self-cells (33) than M2 macrophages and strongly argues for an important functional role of CRT in the phagocytosis process not only as an “eat-me” signal. Notably, CD14 was characterized as a macrophage tethering receptor for apoptotic cells (34, 35); thus, our observation also underlines that this receptor is not uniquely or directly linked to the uptake process but more likely to signaling and/or regulating events.

Why soluble CRT induces a pro-inflammatory signal and a decrease of clearance of cells at the beginning of their death, whereas apoptotic cell membrane-anchored CRT was characterized as an “eat-me” signal, remains to be understood. For instance, we can hypothesize that delaying efferocytosis would help phagocyte reprogramming and also the onset of apoptotic cell-associated molecular patterns to guarantee the adapted immune response to apoptotic cells.

## Author Contributions

PF designed the study and analyzed and interpreted data. RO, PT-D, and AM contributed to the study design and acquired, analyzed, and interpreted data. J-PK contributed to the study design and data interpretations. PF and AM wrote the manuscript. All authors approved the submitted version.

## Conflict of Interest Statement

The authors declare that the research was conducted in the absence of any commercial or financial relationships that could be construed as a potential conflict of interest.
